# A wireless bioresorbable triboelectric system for postoperative pain control

**DOI:** 10.1093/nsr/nwag439

**Published:** 2026-07-16

**Authors:** Justine Lin, Xiujun Fan, Xinkai Xu, Guorui Chen, Jun Chen

**Affiliations:** Department of Bioengineering, University of California, Los Angeles, USA; Department of Bioengineering, University of California, Los Angeles, USA; Department of Bioengineering, University of California, Los Angeles, USA; Department of Bioengineering, University of California, Los Angeles, USA; Department of Bioengineering, University of California, Los Angeles, USA

Postoperative recovery relies on comprehensive perioperative management and patient care, in which pain control plays a critical role [1–4]. Inadequate pain management can lead to delayed recovery, infection, and prolonged patient discomfort. Pharmacological analgesia—particularly opioid-based medication—remains the most common method of pain management [5]. However, growing concerns about opioid addiction, dependence, and systemic side effects have intensified the search for safer alternatives.

Postoperative pain arises from tissue injury and inflammation, which sensitizes the peripheral nervous system and enhances nociceptive signal transmission to the central nervous system. Because pain signals are transmitted through electrically excitable neural pathways, electrical neuromodulation has emerged as a promising strategy for postoperative pain management by interrupting peripheral nerve activity before nociceptive signals reach the central nervous system [6–8]. Compared to traditional pharmacological approaches, electrical stimulation offers localized, reversible, and drug-free pain relief while reducing systemic side effects and the risk of opioid dependence. Several electrical stimulation technologies have been explored for this purpose [9–11]. Transcutaneous electrical nerve stimulation delivers electrical currents through skin-mounted electrodes and is non-invasive, but its limited penetration depth and spatial selectivity restrict effective stimulation of deeper nerves [12]. Percutaneous electrical nerve stimulation involves placing needle electrodes near the target nerve to improve stimulation specificity, although the need for percutaneous access introduces infection risks and limits long-term use [13]. Cuff electrodes, which wrap directly around peripheral nerves and deliver alternating electrical stimulation, provide the most precise neural modulation [14]. However, current cuff-based systems require wired power delivery, bulky external hardware, and often necessitate secondary surgical procedures for device removal.

**Figure 1.**
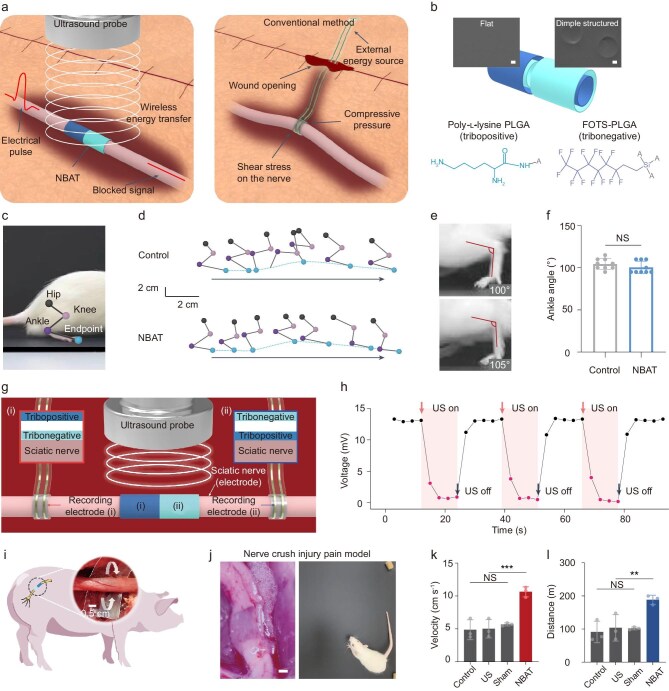
A bioresorbable triboelectric nerve cuff for ultrasound-triggered neuromodulation and pain management. (a) Schematic illustration of the ultrasound-driven NBAT nerve cuff enabling minimally invasive neuromodulation compared with conventional stimulation approaches. (b) Structural design of the NBAT nerve cuff with functional-group modification that enables targeted triboelectric stimulation. Scale bars, 10 μm. (c) Photograph of hindlimb kinetics during walking in rat models. (d) Stick diagram decompositions of hindlimb movements during walking used for gait analysis. (e) Representative photographs of the ankle during the toe-off phase in control and NBAT-treated rats, with the ankle angle indicated. (f) Quantification of ankle angle during the toe-off phase in control animals and rats implanted with the NBAT cuff. (g) Schematic of the experimental configuration used to monitor electrode phase difference during stimulation. (h) Compound nerve action potential recorded before, during, and after repeated ultrasonic activation, measured at 3 s intervals. (i) Schematic illustration of NBAT cuff implantation around the sciatic nerve in a porcine model. Scale bar: 0.5 cm. (j) Left: Photograph of the sciatic nerve crush injury model used to induce acute pain. Scale bar, 1 mm. Right: Open-field behavioral testing setup (40 cm × 40 cm chamber) used to evaluate locomotor activity. (k) Quantification of average movement velocity in control, ultrasound-only (US), sham (NBAT without ultrasound), and NBAT (ultrasound-activated) groups. (l) Total movement distance during a 5 min session. The NBAT group exhibited significantly enhanced locomotor activity compared with controls. Data in (k) and (l) are presented as mean ± s.d.; *n* = 3 biologically independent animals per group, each measured once. Adapted with permission from Ref. [15].

To address these challenges, a recent *Nature Biomedical Engineering* [15] article reports an implantable, biodegradable device that uses triboelectricity to generate a localized electrical modulation around the nerve. This device provides an ultrasound-powered, highly precise, and transient solution that addresses the clinical and mechanical limitations of conventional nerve stimulation systems. It utilizes the Nerve Block System by Alternating Triboelectric-Potential (NBAT) to deliver instantaneous and reversible pain control for acute postoperative pain. Although inspired by kilohertz-frequency alternating current principles, NBAT relies on ultrasound stimulation rather than externally connected bipolar electrodes, reducing invasive materials and stress on the nerve (Fig. 1a). The device consists of two bioresorbable poly(lactic-co-glycolic acid) (PLGA) films with opposite triboelectric polarities generated through surface functionalization (Fig. 1b). Microstructured dimples created by solvent exchange maintain spacing between the films and enable alternating electric field generation during ultrasound-induced vibration. This configuration aligns the electric field parallel to the nerve, perturbing ionic transport across neural membranes and blocking signal propagation. Importantly, the mechanical properties of the polymer layers closely match those of peripheral nerves, minimizing compression and mechanical strain. Moreover, the biodegradable design enables the implant to gradually resorb after therapeutic use, eliminating the need for surgical removal.

To verify the clinical viability of the NBAT, its biocompatibility, mechanical safety, and pain-blocking efficacy were evaluated. Initial *in-vitro* testing demonstrated that fibroblasts cultured on the surface of functionalized PLGA layers showed higher cell viability compared to bare PLGA over 72 h. Subsequently, *in-vivo* studies in rat models confirmed that white blood cell levels remained within normal ranges for 1 month, revealing no detectable immune response at the implantation site even after reabsorption. Beyond biocompatibility, to monitor impact on motor movement—especially if the cuff was wrapped around a motor nerve—a gait analysis was performed (Fig. 1c and d). The rats’ gait height, stride, ankle angles, and toe-off phase were evaluated against parameters from previous studies and showed no

significant differences (Fig. 1e and f). This highlights how the NBAT can conform closely to the nerve without generating the same compression as the commercial cuff, addressing inflammation and nerve damage. To further validate the electrical efficacy of the NBAT, the cuff was wrapped around an electrode with a resistance similar to that of a nerve (Fig. 1g). Upon activation, the output of the NBAT decreased, confirming its ability to influence ion movement (Fig. 1g). In biological models, standard electrophysiological recording techniques monitored how NBAT activation prevented the membrane potential from reaching the threshold required to initiate an action potential (Fig. 1h). Interestingly, in porcine models with larger peripheral nerves, the triboelectric effect increased proportionally with cross-sectional area, further increasing the effectiveness of this device (Fig. 1i). For behavior analysis, activity was assessed using average movement velocity and total travel distance (Fig. 1j). The controls—NBAT without ultrasound activation and ultrasound-only—showed reduced locomotion. By contrast, the NBAT group with ultrasound activation exhibited higher average velocities and travel distances, consistent with an effective suppression of pain-related movement inhibition (Fig. 1k, l). Finally, after ultrasound activation was stopped, compound muscle action potential and compound nerve action potential recovered, demonstrating the reversibility of nerve signaling and lack of damage.

This work introduces a significant shift in acute postoperative pain treatment, offering a sophisticated alternative to both systemic opioid use and rigid, invasive nerve cuffs. By integrating ultrasound energy with biodegradable materials, it successfully addresses key limitations of prevalent clinical strategies by avoiding systemic side effects and minimizing surgery-related patient risks. However, several challenges remain, such as (i) maintaining robustness even if the films shift away the center of the nerve, (ii) the loss of ultrasound wave energy in deeper tissue, (iii) the limited flexibility of the approximately 30 days degradation window, (iv) maintaining stable triboelectric performance in aqueous physiological environments and over extended periods of operation, (v) developing strategies to control the degradation rate to accommodate different postoperative pain durations, and (vi) establishing reliable methods to evaluate pain levels throughout treatment to enable precise neuromodulation. Future research could focus on investigating how tissue depth affects ultrasound wave transmission, especially because human peripheral nerves are significantly deeper than rat or porcine models. Additionally, these approaches could be extended to the management of slower-onset or chronic pain conditions.
